# Supplementary material for: S100A8/9 modulates perturbation and glycolysis of macrophages in allergic asthma mice
Source: PeerJ. 2024 Apr 18;12:e17106. doi: 10.7717/peerj.17106 (PMC11032659; doi:10.7717/peerj.17106)
Supplement: Supplemental Information 1 [file peerj-12-17106-s001.docx]

**Supplemental Figures**


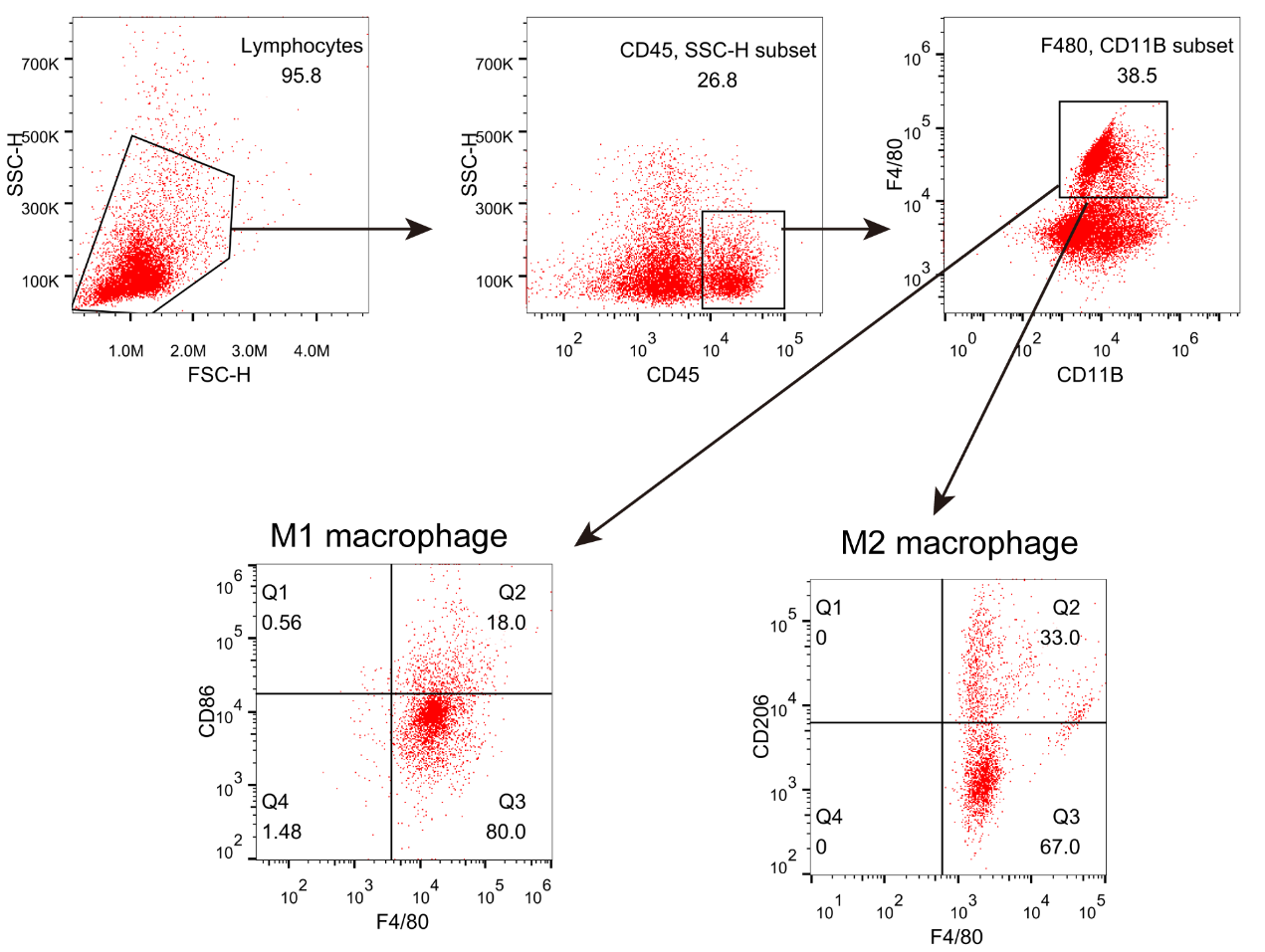


Supplemental Figure 1 Flow cytometry-gating strategy of M1 and M2 macrophages in BALF. Forward scatter (FSC-H) and side scatter (SSC-H) were used to select single cells; Then, SSC-H CD45+ and F4/80+ CD11b+ were used to screen macrophages in that order. CD86+ F4/80+ and CD206+ F4/80+ were used to recognize M1 and M2 macrophages.


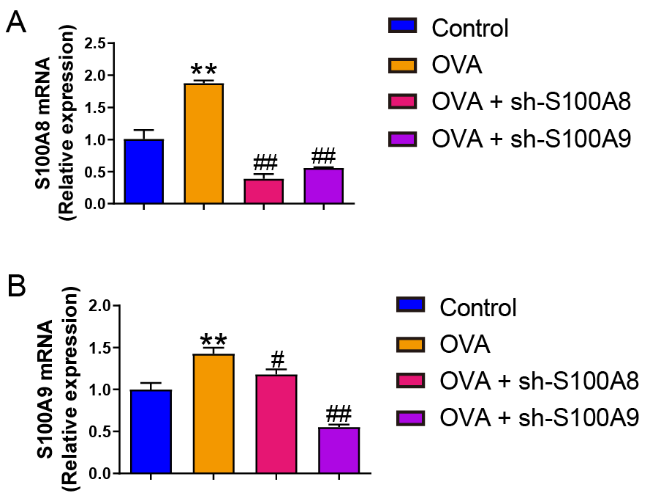


Supplemental Figure 2 Quantitative real-time PCR measured S100A8 and S100A9 mRNA in MH-S cells. S100A8 (A) and S100A9 (B) mRNAs were increased in MH-S cells with OVA intervention, but knockdown of S100A8 or S100A9 antagonized it.


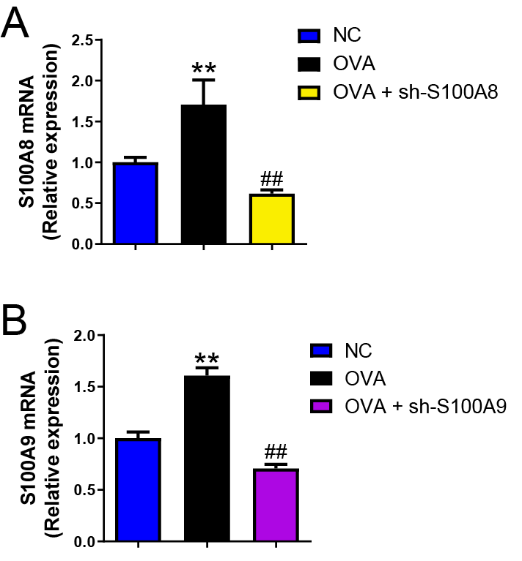


Supplemental Figure 3 Quantitative real-time PCR measured S100A8 and S100A9 mRNA in mice. (A, B) mRNAs of S100A8 and S100A9 were detected by quantitative real-time PCR; they were increased in OVA group and decreased in OVA-induced allergic asthma model mice with S100A8 or S100A9 knockdown.
