# Supplementary figures and images for: S100A8/9 modulates perturbation and glycolysis of macrophages in allergic asthma mice
Source: PeerJ. 2024 Apr 18;12:e17106. doi: 10.7717/peerj.17106 (PMC11032659; doi:10.7717/peerj.17106)

Figure 2N

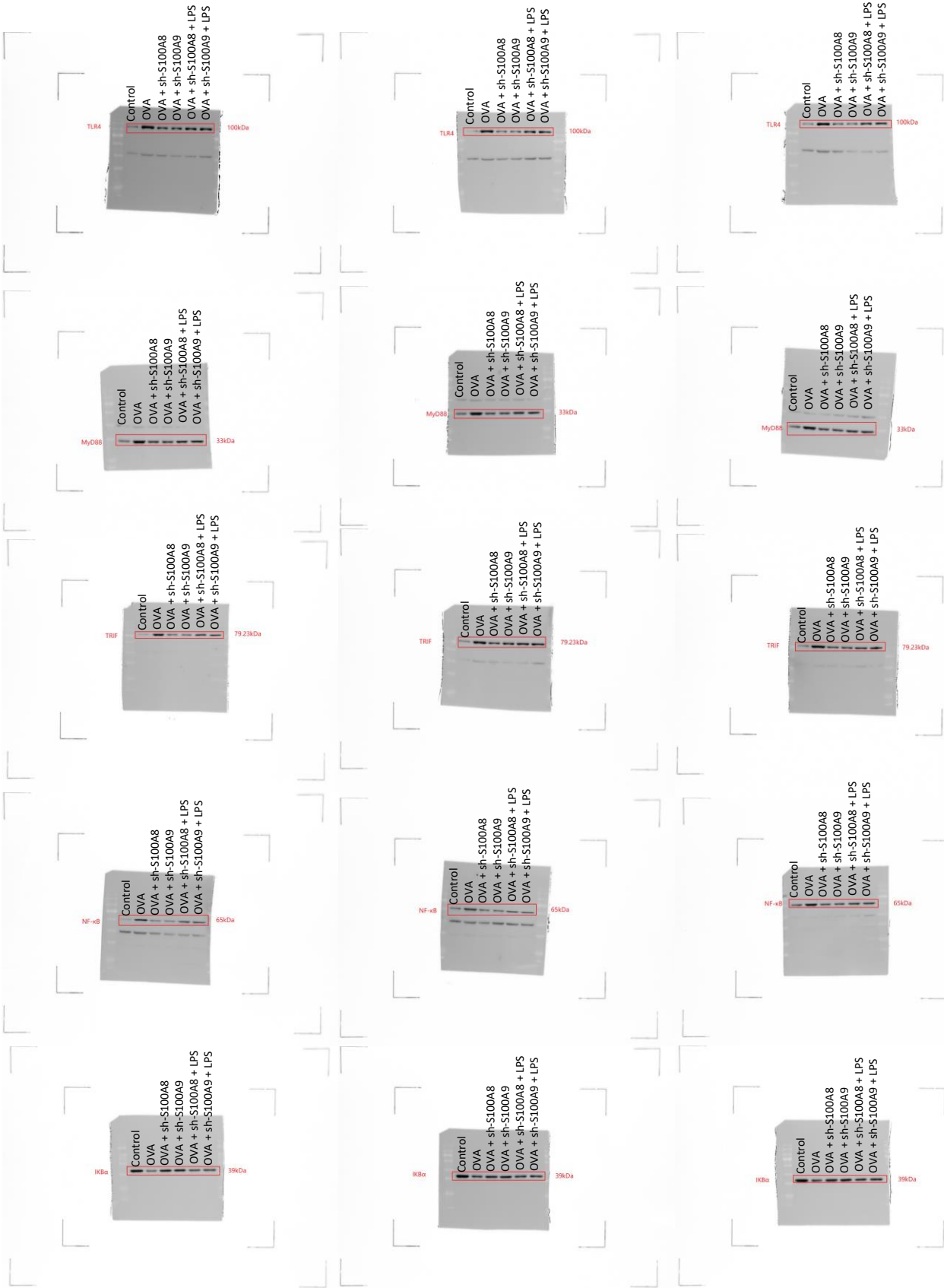

Figure 2N

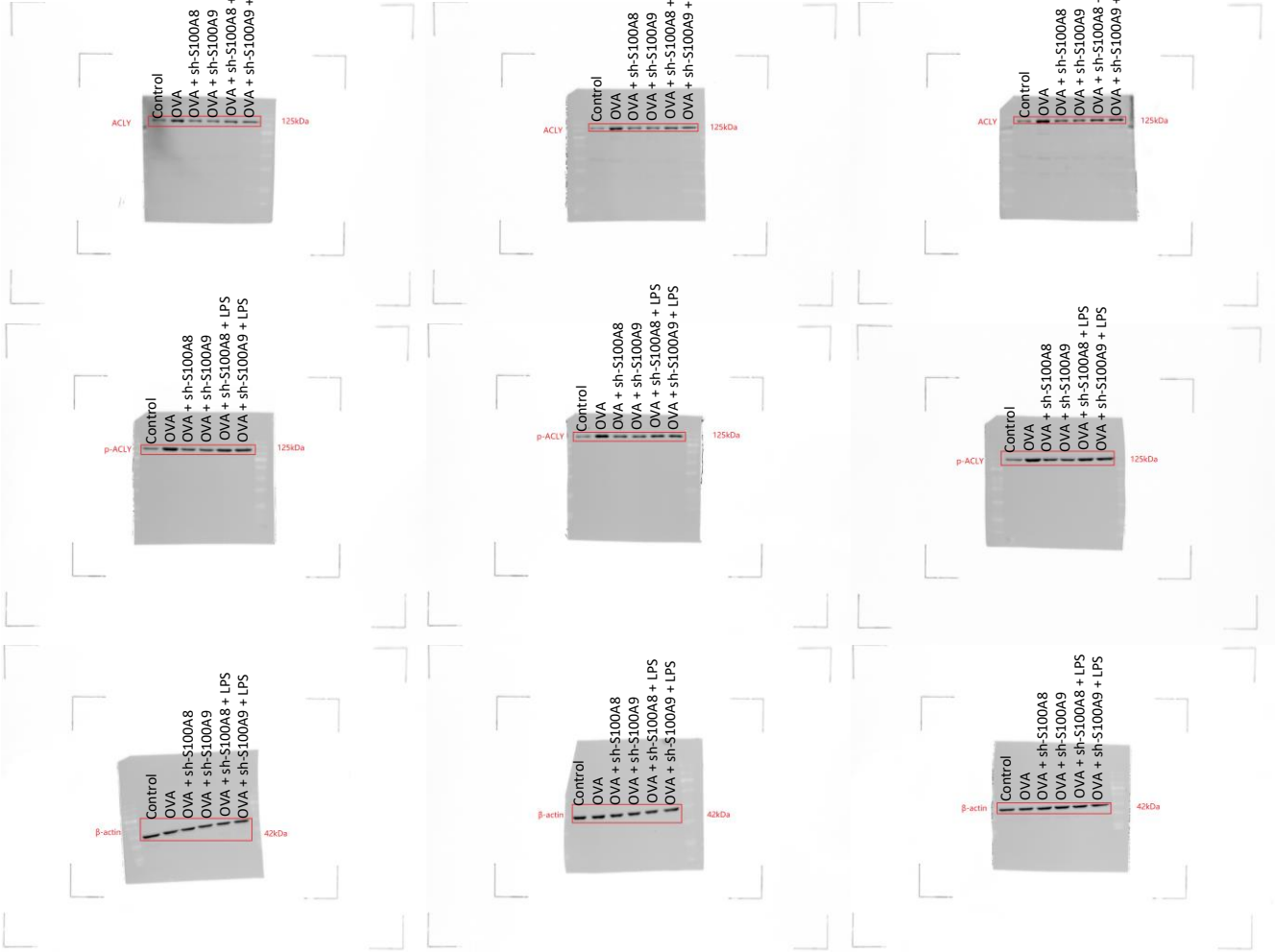

Figure 5M and 8M

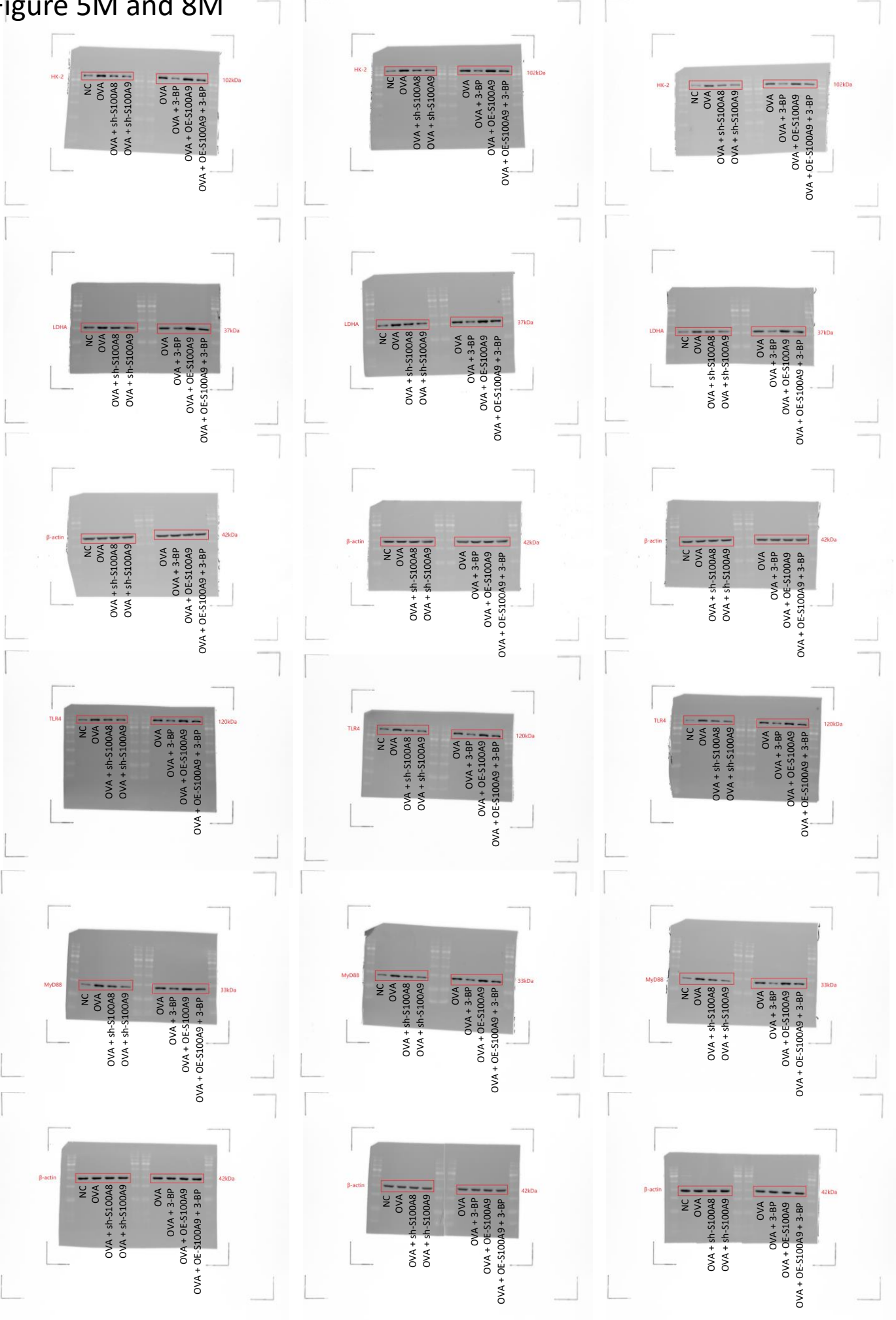

Figure 5M and 8M

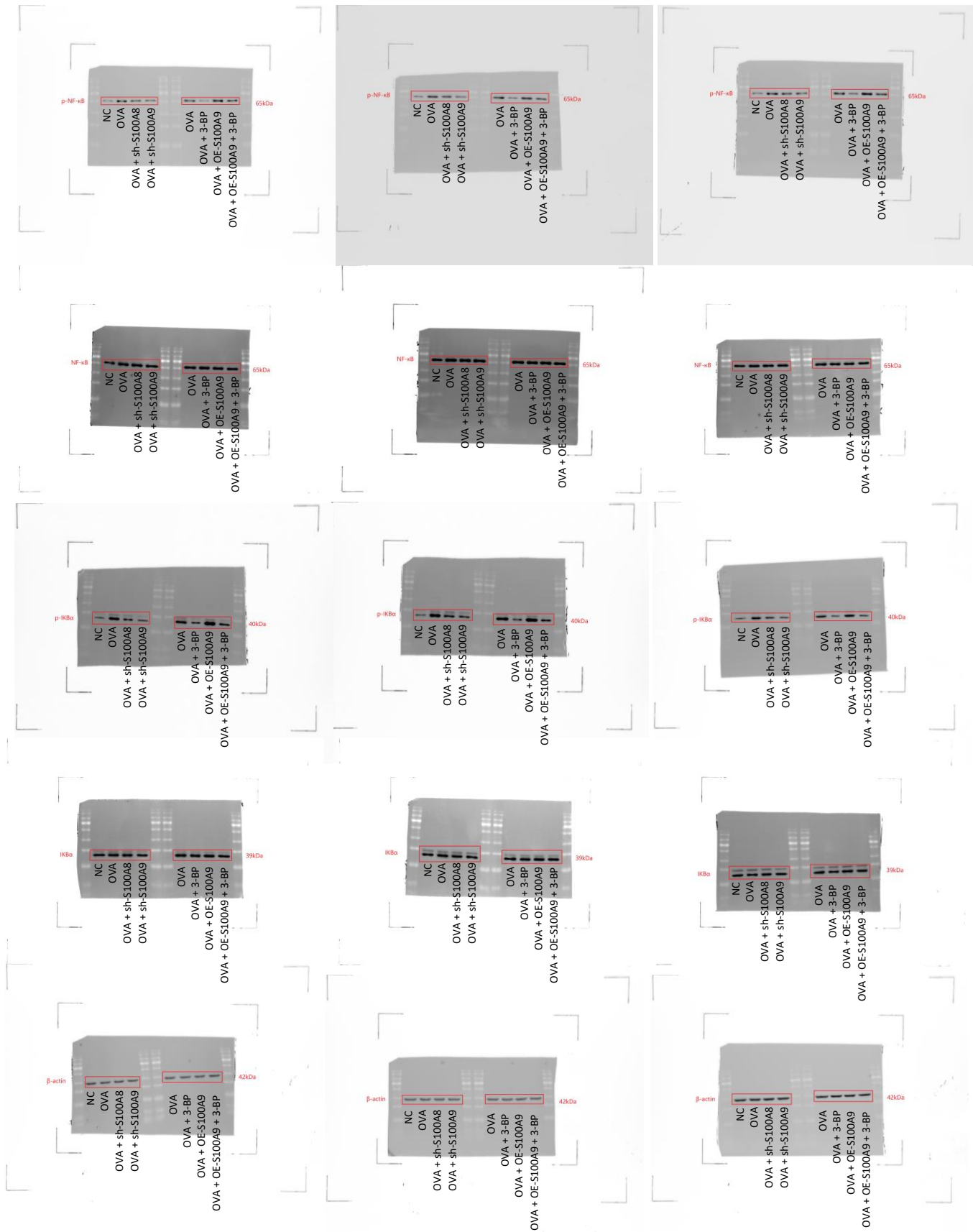

Figure 5M and 8M

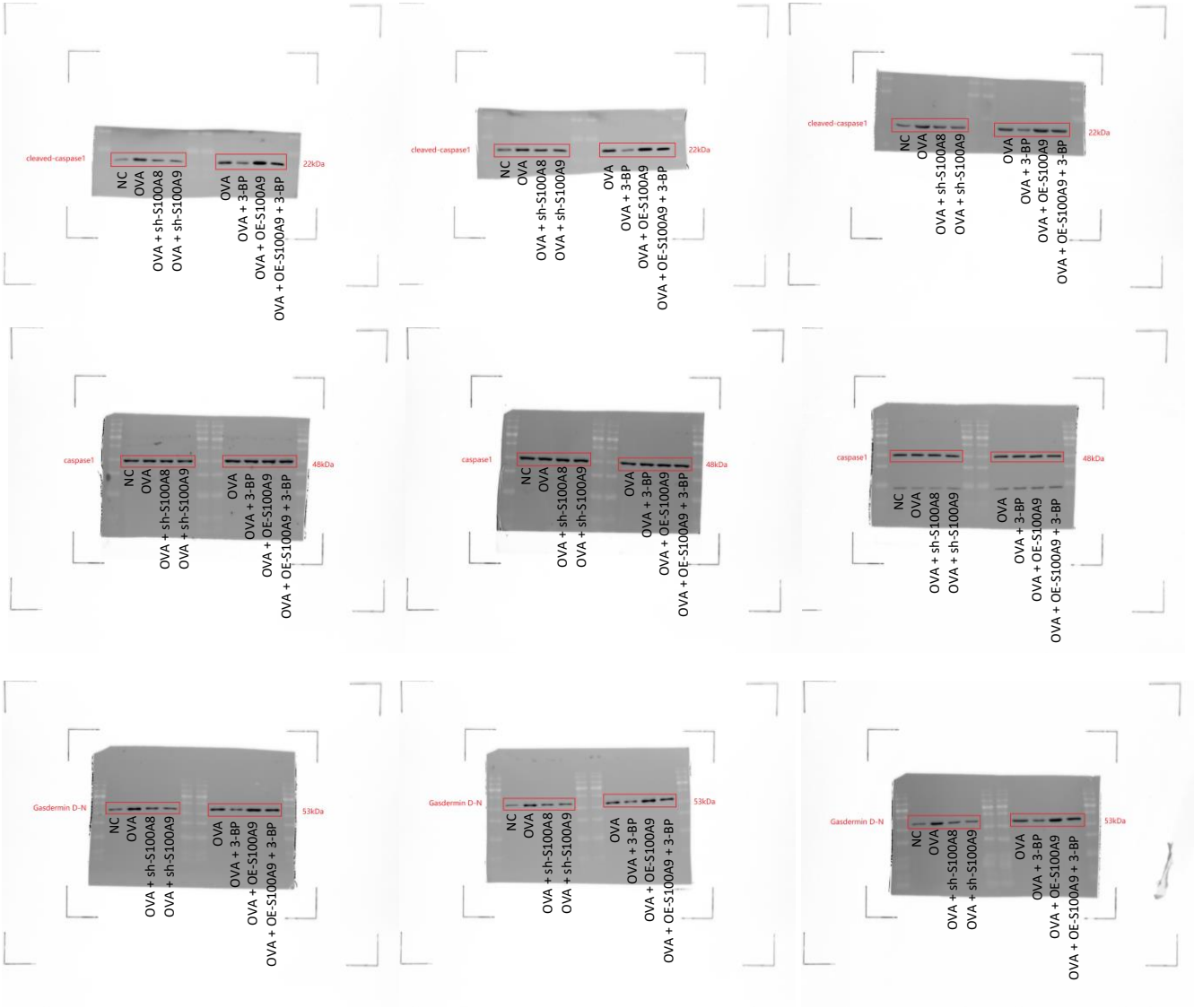

Supplement: Supplemental Information 2 — All protein bands used in Figs. 2, 3, and 8. [file peerj-12-17106-s002.pdf]
